# Prevalence of the root lesion nematode virus (RLNV1) in populations of *Pratylenchus penetrans* from North America

**DOI:** 10.21307/jofnem-2020-045

**Published:** 2020-05-18

**Authors:** Paulo Vieira, Amy Peetz, Benjamin Mimee, Kanan Saikai, Dimitre Mollov, Ann MacGuidwin, Inga Zasada, Lev G. Nemchinov

**Affiliations:** 1 Molecular Plant Pathology Laboratory, USDA-ARS, Beltsville, MD 20705; 2 School of Plant Environmental Science, Virginia Tech, Blacksburg, VA 24061; 3 Horticultural Crops Research Laboratory, USDA-ARS, Corvallis, OR 97330; 4 St-Jean-sur-Richelieu Research and Development Center, Agriculture and Agri-Food Canada, St-Jean-sur-Richelieu, Canada; 5 Department of Plant Pathology, University of Wisconsin–Madison, Madison, WI 53706; 6 National Germplasm Resources Laboratory, USDA-ARS, Beltsville, MD 20705

**Keywords:** Distribution, Picorna-like virus, Pratylenchidae, Variability

## Abstract

Root lesion nematode virus 1 (RLNV1) was discovered in the migratory endoparasitic nematode species *Pratylenchus penetrans*. It was found in a *P. penetrans* population collected from soil samples in Beltsville, Maryland, USA. In this study, the distribution of the RLNV1 in 31 geographically distinct *P. penetrans* populations obtained from different crops was examined. The results demonstrate that RLNV1 is widespread in North American populations of *P. penetrans* and exhibits low genetic variability in the helicase and RNA-dependent RNA polymerase regions of the genome.

Presently, the number of viruses identified in the phylum Nematoda is limited. Considering the vast diversity of species within this phylum, more viruses naturally infecting nematodes are likely to be discovered. Recently, several new viruses were identified from wild populations of free-living (Félix et al., 2011; Franz et al., 2012; Frézal et al., 2019), animal-parasitic (Shi et al., 2016; Williams et al., 2019) and sedentary plant-parasitic nematodes (PPN; Bekal et al., 2011, 2014; Lin et al., 2018; Ruark et al., 2017, 2018).

We have recently discovered a new virus (the root lesion nematode virus, RLNV1) associated with the migratory nematode *Pratylenchus penetrans* (Vieira and Nemchinov, 2019). *P. penetrans* is an endoparasitic migratory PPN, which can infect a broad range of economically important crops (Castillo and Vovlas, 2007) and is among the top three most damaging species of PPN (Jones et al., 2013). *Pratylenchus* species were the most abundant PPN (69%) identified in 38,022 samples from the Pacific Northwest of North America by nematode diagnostic laboratories labs from 2012 to 2016 (Zasada et al., 2019).

The objectives of this study were: to determine the distribution of the RLNV1 in geographically distinct *P. penetrans* populations obtained from different North American cropping systems and to assess genetic variability of the virus by comparing sequence variations of the helicase and RNA-dependent RNA polymerase (RdRP) regions of identified RLNV1 isolates from the *P. penetrans* populations collected for this study.

## Material and methods

### Collection of *Pratylenchus penetrans* isolates

A total of 31 populations of *P. penetrans* were used in this study from different geographic locations across Canada and USA (Table 1). Their identification was confirmed by morphological and molecular markers available for this species (Castillo and Vovlas, 2007; Peetz and Zasada, 2016). Most of the nematode populations were initially collected from different crops and maintained *in vitro* on sterilized corn roots (Vieira et al., 2015). The *P. penetrans* isolate NL 10p RH, from which RLNV1 (GenBank accession MK138531) was first identified, was used as positive control (Vieira and Nemchinov, 2019). For each population, several hundred nematodes were extracted from roots under intermittent mist for 5 d (Ayoub, 1980), washed three times in distilled water, frozen in liquid nitrogen, and stored at −80°C until subsequent analyses.

**Table 1.** Results of the virus detection in different populations of *Pratylenchus penetrans*, and associated host plants.

						Detection and sequencing	
Genus	Species	Population	No. of individuals	Host	Origin of population	Helicase	RdRP
*Pratylenchus*	*penetrans*	NL 10p RH	Bulk	Corn	Beltsville, Maryland	Yes*	Yes*
*Pratylenchus*	*penetrans*	R3605	Bulk	Potato	NA, Michigan	−	−
*Pratylenchus*	*penetrans*	R3606	Bulk	Potato	NA, Michigan	Yes	Yes
*Pratylenchus*	*penetrans*	Greenhouse	200	Mint	Various locations from OR and WA	Yes	Yes
*Pratylenchus*	*penetrans*	Cherry	120	Cherry	Hood River, OR	−	−
*Pratylenchus*	*penetrans*	CAR	200	Apple	Kenniwick, WA	Yes	Yes
*Pratylenchus*	*penetrans*	Pole Road	200	Raspberry	Lynden, WA	−	−
*Pratylenchus*	*penetrans*	R-BDM	Bulk	Raspberry	Lynden, WA	−	−
*Pratylenchus*	*penetrans*	82−13	Bulk	Soybean	Calumet Co., WI	−	−
*Pratylenchus*	*penetrans*	511−14	Bulk	Soybean	Chippewa Co., WI	−	−
*Pratylenchus*	*penetrans*	546−16	Bulk	Soybean	Chippewa Co., WI	Yes	Yes
*Pratylenchus*	*penetrans*	736−13	Bulk	Soybean	Grant Co., WI	Yes	Yes
*Pratylenchus*	*penetrans*	92−16	Bulk	Soybean	Iowa Co., WI	−	−
*Pratylenchus*	*penetrans*	99−16	Bulk	Soybean	Lafayette Co., WI	−	−
*Pratylenchus*	*penetrans*	128−16	Bulk	Soybean	Marathon Co., WI	−	−
*Pratylenchus*	*penetrans*	551−14	Bulk	Soybean	Marquette Co., WI	−	−
*Pratylenchus*	*penetrans*	Malek	Bulk	Potato	Portage CO., WI	Yes	Yes
*Pratylenchus*	*penetrans*	P*	Bulk	Potato	Portage CO., WI	Yes	Yes
*Pratylenchus*	*penetrans*	PRF	Bulk	Potato	Portage CO., WI	Yes	Yes
*Pratylenchus*	*penetrans*	PP6-1	Bulk	Potato	Portage CO., WI	−	−
*Pratylenchus*	*penetrans*	469A-13	Bulk	Soybean	Sheboygan Co., WI	Yes	Yes
*Pratylenchus*	*penetrans*	Lauer	Bulk	Potato	Waushara Co., WI	Yes	Yes
*Pratylenchus*	*penetrans*	422−14	Bulk	Soybean	Wood Co., WI	−	−
*Pratylenchus*	*penetrans*	PA	Bulk	Corn	NA	Yes	Yes
*Pratylenchus*	*penetrans*	R3784	Bulk	Potato	New Brunswick, CA**	No	Yes
*Pratylenchus*	*penetrans*	R3790	Bulk	Potato	New Brunswick, CA**	Yes	Yes
*Pratylenchus*	*penetrans*	R3794	Bulk	Potato	New Brunswick, CA**	Yes	Yes
*Pratylenchus*	*penetrans*	R3813	Bulk	Potato	New Brunswick, CA**	−	−
*Pratylenchus*	*penetrans*	R3816	Bulk	Potato	New Brunswick, CA**	−	−
*Pratylenchus*	*penetrans*	R3739	Bulk	Potato	Prince Edward Island, CA**	−	−
*Pratylenchus*	*penetrans*	R3771	Bulk	Potato	Prince Edward Island, CA**	−	−
*Pratylenchus*	*penetrans*	QCLA	Bulk	Potato	L'Acadie, Quebec, CA**	−	−

**Notes:** NA, not available specific location. *These sequences were original obtained and published in Vieira and Nemchinov (2019); **Canada; the symbol “−” denotes nematode populations with no detection regarding the presence of RLNV1.

### Nematode RNA extraction

Total RNA (50 ng per sample) was extracted from mixed life stages (eggs, second- to fourth-stage juveniles (J2-J4), adult females and males) of *P. penetrans* using the RNeasy Plant Mini Kit (QIAGEN, Hilden, Germany), following the manufacturer’s instructions. RNA was treated with RNase-free DNase (QIAGEN) before reverse transcription. The quantity and quality of the extracted RNA was assessed using a ND-1000 NanoDrop spectrophotometer (Thermo Scientific, Wilmington, DE, USA), and cDNA was synthesized using the iScript cDNA Synthesis Kit (Bio-Rad, Hercules, CA, USA) following the manufacturer’s instructions. Primers were designed based on the available nucleotide sequence of the viral helicase (forward: 5´-GATCTCACGCGCTTTACCA-3´, pos. 4046-4064 and reverse: 5´-TCAGGTTCTGGAACAGGATTTC-3´, pos. 4978-4900) and RdRP (forward: 5´-CCCTATACACAAATGGGAATAACAA-3´, pos. 7329-7353 and reverse: 5´-ATGCTCTCAAACCAGTCACTAT-3´, pos. 8307-8328) and used for PCR amplification of the corresponding sequence regions. The presence of the nematode transcripts within each generated cDNA library was confirmed by amplification of the 18 S rRNA gene fragment of *P. penetrans* (forward: 5´-CGTAAGGGAAGAGCGCATTTA-3´ and reverse primers: 5´-CAGATACCCTACCATCGAAAGTT-3´). Reverse transcription-polymerase chain reaction (RT-PCR) was conducted using 1 μl of cDNA from each library and the following conditions: 2 min at 94°C; 38 cycles (30 sec at 94°C, 30 sec at 54°C, 60 sec at 72°C), and one cycle at 72°C for 10 min. The PCR reactions contained 1 × PCR buffer, 1 unit Taq Platinum polymerase (Invitrogen, Carlsbad, CA, USA) and 0.2 μM of each primer in a total of 50 μl of total solution. PCR products were separated by electrophoresis on a 1% agarose gel using TBE buffer (0.045 M Trisborate, 0.001 M EDTA, pH 8.0) and visualized using SYBR Safe DNA gel stain (Invitrogen, Carlsbad, CA, USA). The generated PCR products were then purified by PCR-purification kit (QIAGEN) and sequenced by Sanger sequencing using the corresponding forward and reverse primers by Macrogen Corp (Rockville, MD, USA).

### Analysis of genetic diversity

Nucleotide sequences were aligned using MUSCLE program with default parameters incorporated into CLC Main Workbench software (V. 8). Predicted proteins sequences were obtained using CLC Main Workbench software (V. 8) and aligned using MUSCLE program with default parameters (Edgar, 2004). Pairwise genetic distances of both nucleotide and amino acid sequences (i.e. nucleotide differences and percent identity) were determined using CLC Main Workbench V. 8 (Qiagen, Hilden, Germany) software.

## Results

### Geographic distribution of RLNV1

**Figure 1:**
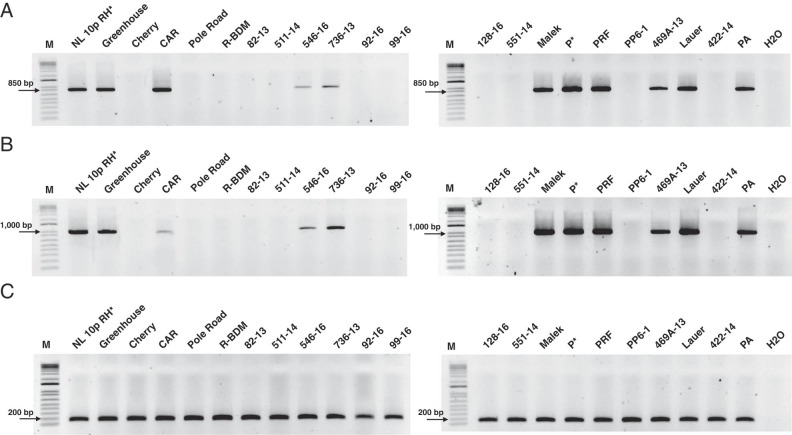
Reverse transcription-polymerase chain reaction (RT-PCR) detection of the root lesion nematode virus (RLNV1) from cDNA libraries generated from 21 out of the 31 populations of *Pratylenchus penetrans* collected in North America. PCR amplification was performed using specific primers for the RLNV1 helicase (A) and RdRP (B) regions. The isolate NL 10p RH was used as positive control for the detection of the RNLV1. C: The nematode 18 S rRNA gene was used as a positive control to validate the presence of *P. penetrans* transcripts within each generated cDNA library.

A total of 31 populations of *P. penetrans* were assessed for the presence of the RLNV1 by RT-PCR with primers derived from helicase and RdRP regions of the virus genome (Fig. 1 and Table 1). These isolates were obtained from either established cultures or field collections of *P. penetrans*, originally collected from agricultural fields distributed throughout several areas of the USA and Canada (Table 1). *Pratylenchus penetrans* populations were collected mainly from potato and soybean fields, but were also collected from apple, cherry, corn, mint, and raspberry, which is consistent with the wide host range of this nematode species (Castillo and Vovlas, 2007).

PCR amplification resulted in amplicons of the expected sizes (855 bp for the helicase and 1,000 bp for the RdRP regions) from 14 out of the 31 *P. penetrans* populations (Fig. 1A, B). The presence of *P. penetrans* transcripts within each cDNA library was confirmed by amplification of a 150 bp fragment from the 18 S rRNA gene (Fig. 1C). The RLNV1 was found in *P. penetrans* populations collected from potato, soybean, mint, apple and corn fields, while the virus was not detected in *P. penetrans* populations obtained from raspberry and cherry (Table 1). Overall, the RLNV1 was detected in 45% of the *P. penetrans* populations.

### Sequence variability within RLNV1 isolates

The partial nucleotide sequences of the helicase and RdRP regions derived from 14 RLNV1 isolates associated with different *P. penetrans* populations were obtained and compared between each other (Tables S1 and S2). In one population (*P. penetrans* population R3784 collected from potato in New Brunswick, Canada), only the RdRP region was sequenced. Sequences exhibited a low level of nucleotide and amino acid variations in both regions of the viral genome. Pairwise comparisons of the nucleotide sequences in the helicase region ranged from 96.56 to 100%, while at the amino acid level the corresponding sequences had 98.89 to 100% identity among all the isolates (Table S1). For the RdRP fragment of the genome, the sequence identity ranged from 96.75 to 100% at the nucleotide level, and 98.11 to 100% at the amino acid level (Table S2). The Malek isolate originally collected from a potato field in Wisconsin displayed the highest genetic variability among all isolates.

**Table S1.** Nucleotide (A) and protein (B) pairwise comparisons of the helicase sequence data of different RLNV1 isolates from *Pratylenchus penetrans*.

A														
	RLNV1	3606	Green house	Car	546-16	736-13	Malele	P*	PRF	469A	Lauer	PA	R3794	R3790
RLNV1		98.65	98.77	99.26	98.53	98.89	96.93	98.89	99.63	98.89	98.65	99.75	98.65	99.63
3606	11		99.39	99.14	99.14	99.51	96.56	99.51	99.02	99.51	99.75	98.89	99.75	99.02
Greenhouse	10	5		99.26	99.26	99.63	96.93	99.63	99.14	99.63	99.39	99.02	99.39	99.14
Car	6	7	6		99.26	99.63	97.17	99.63	99.63	99.63	99.14	99.51	99.14	99.63
546-16	12	7	6	6		99.63	96.44	99.63	98.89	99.63	99.14	98.77	99.14	98.89
736-13	9	4	3	3	3		96.81	100	99.26	100	99.51	99.14	99.51	99.26
Malele	25	28	25	23	29	26		96.81	97.3	96.81	96.56	97.17	96.56	97.3
P*	9	4	3	3	3	0	26		99.26	100	99.51	99.14	99.51	99.26
PRF	3	8	7	3	9	6	22	6		99.26	99.02	99.88	99.02	100
469A	9	4	3	3	3	0	26	0	6		99.51	99.14	99.51	99.26
Lauer	11	2	5	7	7	4	28	4	8	4		98.89	99.75	99.02
PA	2	9	8	4	10	7	23	7	1	7	9		98.89	99.88
R3794	11	2	5	7	7	4	28	4	8	4	2	9		99.02
R3790	3	8	7	3	9	6	22	6	0	6	8	1	8	
B														
	RLNV1	3606	Green house	Car	546-16	736-13	Malele	P*	PRF	469A	Lauer	PA	R3794	R3790
RLNV1		99.63	100	100	99.26	100	100	100	100	100	100	100	100	100
3606	1		99.63	99.63	98.89	99.63	99.63	99.63	99.63	99.63	99.63	99.63	99.63	99.63
Greenhouse	0	1		100	99.26	100	100	100	100	100	100	100	100	100
Car	0	1	0		99.26	100	100	100	100	100	100	100	100	100
546-16	2	3	2	2		99.26	99.26	99.26	99.26	99.26	99.26	99.26	99.26	99.26
736-13	0	1	0	0	2		100	100	100	100	100	100	100	100
Malele	0	1	0	0	2	0		100	100	100	100	100	100	100
P*	0	1	0	0	2	0	0		100	100	100	100	100	100
PRF	0	1	0	0	2	0	0	0		100	100	100	100	100
469A	0	1	0	0	2	0	0	0	0		100	100	100	100
Lauer	0	1	0	0	2	0	0	0	0	0		100	100	100
PA	0	1	0	0	2	0	0	0	0	0	0		100	100
R3794	0	1	0	0	2	0	0	0	0	0	0	0		100
R3790	0	1	0	0	2	0	0	0	0	0	0	0	0	

Note: Lower and upper panels represent the number of different nucleotides (counts) and nucleotide sequence similarity (%) within isolates, respectively.

**Table S2.** Nucleotide (A) and protein (B) pairwise comparisons of the RdRP sequence data of different RLNV1 isolates from *Pratylenchus penetrans*.

A															
	RLNV1	3606	Green house	Car	546-16	736-13	Malele	P*	PRF	469A	Lauer	PA	R3784	R3790	R3794
RLNV1		99.16	99.16	98.74	98.95	98.85	97.9	98.85	99.69	98.95	99.27	99.79	98.43	98.85	98.64
3606	8		99.58	98.95	99.16	99.06	97.48	99.06	99.48	99.16	99.69	99.16	99.06	98.64	99.27
Greenhouse	8	4		99.37	99.58	99.48	97.48	99.48	99.48	99.58	99.48	99.16	98.85	98.64	99.06
Car	12	10	6		99.79	99.69	97.06	99.69	99.06	99.79	98.85	98.74	98.22	98.22	98.43
546-16	10	8	4	2		99.9	97.27	99.9	99.27	100	99.06	98.95	98.43	98.43	98.64
736-13	11	9	5	3	1		97.17	99.79	99.16	99.9	98.95	98.85	98.32	98.32	98.53
Malele	20	24	24	28	26	27		97.17	98.01	97.27	97.38	97.69	96.75	97.17	96.96
P*	11	9	5	3	1	2	27		99.16	99.9	98.95	98.85	98.32	98.32	98.53
PRF	3	5	5	9	7	8	19	8		99.27	99.37	99.69	98.74	99.16	98.95
469A	10	8	4	2	0	1	26	1	7		99.06	98.95	98.43	98.43	98.64
Lauer	7	3	5	11	9	10	25	10	6	9		99.27	98.95	98.53	99.16
PA	2	8	8	12	10	11	22	11	3	10	7		98.43	98.85	98.64
R3784	15	9	11	17	15	16	31	16	12	15	10	15		99.37	99.79
R3790	11	13	13	17	15	16	27	16	8	15	14	11	6		99.37
R3794	13	7	9	15	13	14	29	14	10	13	8	13	2	6	
B															
	RLNV1	3606	Green house	Car	546-16	736-13	Malele	P*	PRF	469A	Lauer	PA	R3784	R3790	R3794
RLNV1		99.37	99.68	99.68	99.68	99.68	99.37	99.37	99.68	99.68	100	100	98.42	98.11	98.42
3606	2		99.68	99.68	99.68	99.68	99.37	99.37	99.68	99.68	99.37	99.37	98.42	98.11	98.42
Greenhouse	1	1		100	100	100	99.68	99.68	100	100	99.68	99.68	98.74	98.42	98.74
Car	1	1	0		100	100	99.68	99.68	100	100	99.68	99.68	98.74	98.42	98.74
546-16	1	1	0	0		100	99.68	99.68	100	100	99.68	99.68	98.74	98.42	98.74
736-13	1	1	0	0	0		99.68	99.68	100	100	99.68	99.68	98.74	98.42	98.74
Malele	2	2	1	1	1	1		99.37	99.68	99.68	99.37	99.37	98.42	98.11	98.42
P*	2	2	1	1	1	1	2		99.68	99.68	99.37	99.37	98.42	98.11	98.42
PRF	1	1	0	0	0	0	1	1		100	99.68	99.68	98.74	98.42	98.74
469A	1	1	0	0	0	0	1	1	0		99.68	99.68	98.74	98.42	98.74
Lauer	0	2	1	1	1	1	2	2	1	1		100	98.42	98.11	98.42
PA	0	2	1	1	1	1	2	2	1	1	0		98.42	98.11	98.42
R3784	5	5	4	4	4	4	5	5	4	4	5	5		99.68	100
R3790	6	6	5	5	5	5	6	6	5	5	6	6	1		99.68
R3794	5	5	4	4	4	4	5	5	4	4	5	5	0	1	

**Note:** Lower and upper panels represent the number of different nucleotides (counts) and nucleotide sequence similarity (%) within isolates, respectively.

## Discussion

The main objective of this study was to characterize the extent of RLNV1 infection in *P. penetrans* populations collected from different plant hosts across North America. We conclude that the virus is widespread in the USA and Canada and affects *P. penetrans* populations collected from the diverse crop systems in North America. However, the virus was not found in *P. penetrans* collected from 11 geographic locations (Fig. 1 and Table 1) and in the nematodes collected from raspberries and cherries (Table 1).

These findings may potentially indicate the presence of virus-free or virus-resistant *P. penetrans* populations, especially in the case of positive and negative results in nematodes collected from the same crop (potato and soybean) and in contiguous geographic locations. Negative results obtained with *P. penetrans* populations collected from raspberry and cherry may also suggest host-dependent susceptibility of *P. penetrans* to RLNV1. If true, this would likely to be related to the genetic variability among the nematode isolates rather than to the virulence of RLNV1, which exhibited considerable homogeneity in the two regions examined in this study.

The observed prevalence of RLNV1 in populations of *P. penetrans* may also imply that this virus could represent a new resource as a potential biological control agent. Viruses associated with *C. elegans* (Orsay virus) and *C. briggsae* (Santeuil Le Blanc and Melnik viruses) were shown to infect intestinal cells, were horizontally transmitted, and slowed host progeny, thus affecting host fitness (Félix et al., 2011; Félix and Wang, 2019). While the interaction of RLNV with *P. penetrans* and its impact on the nematode’s viability and parasitism are not well understood, this approach may shed light on the potential avenue to reduce damage caused by *P. penetrans* to crop plants.
